# Self-Assembly of Small Organic Molecules into Luminophores for Cancer Theranostic Applications

**DOI:** 10.3390/bios12090683

**Published:** 2022-08-25

**Authors:** Jing Wang, Xueliang Wang, Kai Yang, Sijun Hu, Wanhe Wang

**Affiliations:** 1 Research & Development Institute of Northwestern Polytechnical University in Shenzhen, 45 South Gaoxin Road, Shenzhen 518057, China; 2 Institute of Medical Research, Northwestern Polytechnical University, 127 West Youyi Road, Xi’an 710072, China; 3 Collaborative Innovation Center of NPU, Shanghai 201100, China; 4 Chongqing Technology Innovation Center, Northwestern Polytechnical University, Chongqing 400000, China; 5 School of Chemistry and Chemical Engineering, Shandong University of Technology, Zibo 255049, China

**Keywords:** self-assembly, theranostic, cancer, peptide, amphiphilic, aggregation-induced emission

## Abstract

Self-assembled biomaterials have been widely explored for real-time fluorescence imaging, imaging-guided surgery, and targeted therapy for tumors, etc. In particular, small molecule-based self-assembly has been established as a reliable strategy for cancer theranostics due to the merits of small-sized molecules, multiple functions, and ease of synthesis and modification. In this review, we first briefly introduce the supramolecular chemistry of small organic molecules in cancer theranostics. Then, we summarize and discuss advanced small molecule-based self-assembly for cancer theranostics based on three types, including peptides, amphiphilic molecules, and aggregation-induced emission luminogens. Finally, we conclude with a perspective on future developments of small molecule-based self-assembled biomaterials integrating diagnosis and therapy for biomedical applications. These applications highlight the opportunities arising from the rational design of small organic molecules with self-assembly properties for precision medicine.

## 1. Introduction

Cancer is one of the leading causes of death worldwide, posing a heavy economic burden on every country [1]. According to GLOBOCAN 2020, new cancer cases were estimated to be 19.3 million, while cancer death cases reached almost 10.0 million [2]. In addition to the surgical removal of tumors, the most common methods of treating cancer are chemotherapy and radiation therapy, but these treatments generally have serious side effects, such as systemic toxicity and adverse resistance to chemotherapy drugs. A deep understanding of the tumor microenvironment inspires researchers to apply stimuli-responsive drugs to better target tumor tissues and reduce the side effects [3]. In recent decades, with the extensive exploration of integrated diagnostic and therapeutic nanoreagents, nanotheranostics have shown great promise for the treatment of cancer by targeted delivery, with reduced systemic toxicity, controllable release, and multiple therapies for highly efficient anticancer therapy [4].

At the same time, fluorescence imaging has also attracted tremendous attention for biological application due to its salient merits of high sensitivity, real-time and non-invasive manner, and facile equipment [5,6,7,8,9,10]. Surgical resection of tumors can sometimes lead to unclean or excessive resection, resulting in a high possibility of tumor recurrence and seriously affecting the therapeutic effect; therefore, methods of differentiating cancerous tissues from normal tissues will help surgeons to operate more smoothly and accurately [11,12]. With the development of these advanced technologies, multimodal systems combining imaging and drug therapy have become the focus of interest due to their theranostic functions [13,14]. However, the design of theranostic probes generally involves the modification of nanomaterials or conjugation of fluorescent probes to bioactive drugs, which involves a complicated system, and it is hard to effectively control their cellular and in vivo behaviors [15].

Molecular self-assembly is to make use of specific molecular recognition between molecules or between a fragment of a molecule and another fragment of a molecule to form a supermolecular structure through non-covalent interaction [16,17,18]. Self-assembly of molecules is generally driven by the spontaneous synergy of weak interactions based on numerous non-covalent bonds, such as hydrogen bonding, electrostatic interactions, and hydrophobic interactions [19]. For amphiphilic compounds, the hydrophobic effect is the main driving force of self-assembly, and the morphology of self-assembly products can be well adjusted by the proportion of hydrophilic and hydrophobic segments [20]. Self-assembly also can be triggered by hydrogen bonding, which is well known in DNA base pairing, and this type of self-assembly is commonly used to form hydrogels for biomedical applications [21]. In addition, metal coordination also serves as an important driving force, which commonly exists in the self-assembly of metal polydentate ligands [22]. To obtain a more precise and well-ordered self-assembly system, it generally involves multi-level self-assembly, which often occurs in complex organisms [23]. 

Inspired by extensive efforts in self-assembly materials and the self-assembly phenomenon in organisms, numerous self-assembly molecules have been applied for real-time imaging, optical treatment function, and tumor-targeted therapy [24,25,26]. In particular, the self-assembly of small molecules—including peptides, amphiphilic molecules, and aggregation-induced emission luminogens (AIEgens)—offers great opportunities for developing smart theranostic probes for cancers and other diseases, benefiting from the advantages of the ease of synthesis, modular properties, and good biocompatibility [18]. Although various reviews have reported on the self-assembly of molecules, most of them were based on self-assembled nanomaterials or a summary of a single function, and only a few of them focused on the application of self-assembly materials in the integration of diagnosis and treatment [27,28,29]. In this review, we take the supramolecular chemistry of small organic molecules for theranostics as the entry point and summarize the applications of small organic molecule-based self-assembled nanomaterials in cancer theranostics, which contains peptide-, amphiphilic molecule-, and AIEgen-based assembly. We believe that this review will help researchers better understand small organic molecule-based self-assembly for simultaneous cancer diagnosis and treatment.

## 2. Self-Assembly of Small Organic Molecules in Theranostics

Small molecules have desirable advantages for theranostics, such as their small size to better penetrate solid tumor tissues and the ease of synthesis and modification [14]. Taking advantage of these characteristics of small molecules, a strategy of strengthening the donor–acceptor conjugation for increasing intramolecular motions renders the molecule with a high photothermal conversion efficiency value of 80% under 1064 nm laser irradiation [30]. Therefore, small molecules can be easily tailored for biomedical applications. The tumor microenvironment is always slightly different from the normal in vivo microenvironments, with the characteristics of low oxygen and low pH [31]. So it is very important to monitor the microenvironment abnormality in the early stage of the tumor for timely intervention, avoiding the further progression and metastasis of cancer. Recently, dual lock-and-key fluorescent probes have been developed for the precise imaging of tumors in mice models, indicating the potential of small molecule-based fluorescent probes for precision diagnosis of cancer [32,33]. 

On the other hand, with the widespread use of anticancer drugs, whether from the perspective of the overall development of anticancer drugs or the perspective of precise individual medication, it is necessary to mention the topics related to drug resistance [34]. Currently, the main way to solve this problem is to synergistically act on cancer cells through drug combinations [35]. In particular, the strategy of assembly provides a reliable method for combining several bioactive compounds into one system. For example, the co-assembly of a 10-hydroxycamptothecine (HCPT)-peptide amphiphile and the positively charged *cis*-platin achieved efficient nuclear accumulation for combating drug resistance. [36]. It is well accepted that metastasis of malignant tumors is often the main cause of tumor therapy failure. [37]. Recently, a study showed that heparan sulfate (HS)-instructed self-assembled peptides hinder the interaction between heparin-binding EGF-like growth factor (HB-EGF) and heparan sulfate proteoglycans (HSPG), thus inhibiting cancer cell migration [38]. it is therefore an effective way to inhibit the metastasis of malignant tumors and inspires researchers to explore therapeutics by using the self-assembly of small molecules. Furthermore, although small molecules have been widely applied in the field of medicine, they suffer from the problem of short retention in tumor tissues because of their smaller structure providing easy clearance [39]. While self-assembly of small molecules enlarges their size and shape, which enhances their retention time for better therapeutic outcomes, their applications in the medical field can be largely expanded. 

Based on the considerations above, the self-assembly of small molecules has been considerably employed as an advanced nanosystem for cancer theranostics due to their unique all-in-one capacity and other additional functions [40]. We will mainly summarize and discuss applications of three types of self-assembly of small organic molecules, including peptides, amphiphilic molecules, and AIEgens, in the following sections. 

## 3. Peptide-Based Assembly for Theranostic Applications

Since the clinical use of insulin [41], peptide-based therapeutic agents have received intensive attention because of their high specificity, efficacy, and safety [42]. Over the past decade, the peptide-based assembly has developed by leaps and bounds since scientists have taken inspiration from the fact that proteins form a wide variety of functional structures in organisms [43]. Different kinds of amino acids with various structures, permutations, and combinations, as well as high diversity, can be realized during self-assembly for disease diagnosis and treatment [29]. All in all, the most important advantages of peptides as part of self-assembled biomaterials are their biological compatibility and specificity for cancer theranostics (Table 1).

**Figure 1:**
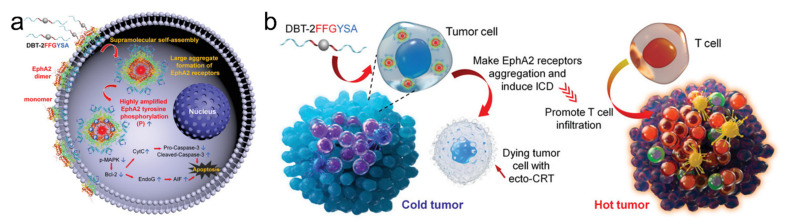
(**a**) Schematic diagram of DBT-2FFGYSA inducing EphA2 receptors form large aggregates beyond dimers. (**b**) Schematic diagram of the proposed mechanism for converting immunologically cold to hot tumors by DBT-2FFGYSA. Adapted with permission from [47]. Copyright 2021 John Wiley and Sons.

By utilizing the structural advantages of natural amino acids and the targeted localization of peptides, in-depth studies on the peptide-based assembly have been carried out [44]. Certain peptide sequences can act as ligands that target specific proteins in tumor cells; for example, pillar [5] arene-based host–guest recognition is used to construct a supramolecular peptide by use of bioactive peptide sequences. Due to the ERGDS sequences on the exterior surfaces and hydrophobic cores of the self-assemblies, the nanoaggregates assembled from the peptides are suitable vehicles to encapsulate a photosensitizer for photodynamic therapy (PDT) [45]. A recent study reported a programmable peptide molecule, consisting of a dual CD3 and integrin α_v_β_3_ targeting sequence (antiCD3-G7-RGD), for the specific recognition of the CD3 receptor on T cells, thus inducing T cell-mediated cytolysis against integrin α_v_β_3_-overexpressing tumor cells [46]. The peptides were labeled with an environment-sensitive fluorophore, 4-nitro-2,1,3-benzoxadiazole (NBD), providing the visualization of peptide behaviors during self-assembly. In both cases, a peptide sequence of RGD was used, which selectively targeted the α_v_β_3_ receptor, endowing the assembled nanoparticles with specificity to cancer cells. Two peripheral peptides, FFGYSAYPDSVPMMS (FFGYSA), were used to promote self-assembly and improve targeting [47], in which YSA selectively targeted tumors that overexpressed the EphA2 protein receptor, while the environmentally sensitive fluorescent molecule was used to generate the fluorescence signal (Figure 1). The peptides made EphA2 receptors form large aggregates beyond dimers, inducing immunogenic cell death (ICD) along with the fluorescence signal.

**Figure 2:**
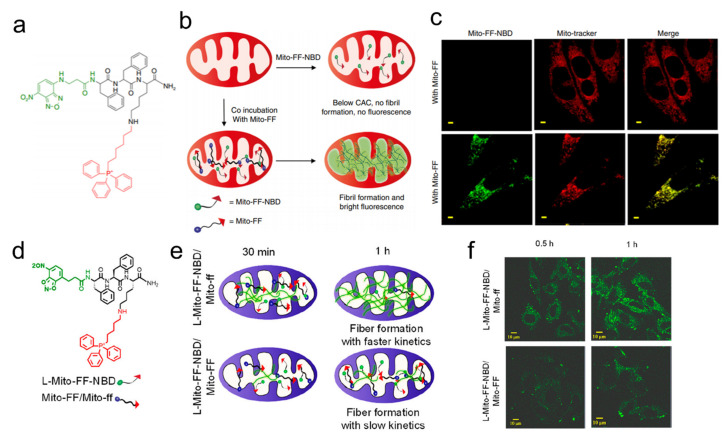
(**a**) Structural design of the mitochondria-targeting peptide amphiphile, Mito-FF. (**b**) Schematic diagram of co-assembly of Mito-FF-NBD with Mito-FF inside mitochondria. (**c**) Co-assembly inside mitochondria indicated by the bright green fluorescence of Mito-FF-NBD in the presence of Mito-FF. (**d**) Structural design of L-Mito-FF-NBD. (**e**) Schematic diagram of the racemic co-assembly of L-Mito-FF-NBD with Mito-ff. (**f**) Time-dependent confocal imaging after co-incubation of L-Mito-FF-NBD/Mito-FF or L-Mito-FF-NBD/Mito-ff. Adapted with permission from [50]. Copyright 2017 Springer Nature. Adapted with permission from [51]. Copyright 2019 American Chemical Society.

Mitochondria is a promising organelle in light of its crucial cellular functions [48]. Triphenyl phosphinium (TPP) has been routinely used for targeting mitochondria and enzyme-instructed self-assembly for cell targeting [18]. In 2016, as the first example of the integration of cell and subcellular targeting, a study designed the molecules to selectively kill cancer cells by inducing dysfunction of mitochondria to release cytochrome c [49]. A tetrapeptide, Phe-Phe-Tyr-Lys (FFYK), was used for achieving self-assembly, along with the use of NBD as a fluorescent reporter for monitoring the self-assembly process in the cellular milieu. Similarly, Ryu et al. designed a similar tripeptide, Phe-Phe-Lys (FFK), which self-assembled to form nanofibers in mitochondria (Figure 2a–c) [50]. The backbone and *N*-terminus of FFK were conjugated with pyrene butyric acid, which not only served as a fluorophore but also drove self-assembly by enhancing hydrophobic and π–π interactions (Figure 2d–f) [51]. Further experiments finally concluded that the co-administration of peptides containing both L- and D-isomers (Mito-FF and Mito-ff) can lead to the cell-induced drastic mitochondrial disruption both in vitro and in vivo. This work provided a general strategy of selectively killing cancer cells through inducing mitochondrial dysfunction along with visualization. In 2018, an enzymatically generated crescent-shaped supramolecular assembly of short peptides was designed for disrupting cell membranes and endoplasmic reticulum (ER) for selective cancer cell death, which employed NBD for fluorescently monitoring the assemblies’ accumulation of peptides in ER [52]. Besides targeting the mitochondria, Golgi is also a potential organelle for targeting because one of its proteins, progestin and adipoQ receptor 11 (PAQR11), is regulated by *TP53* for promoting tumor metastasis [53]. The replacement of an oxygen atom of the phosphoester bond in a phosphopeptide with a sulfur atom enabled the assemblies to quickly target Golgi and selectively kill cancer cells [54].

**Figure 3:**
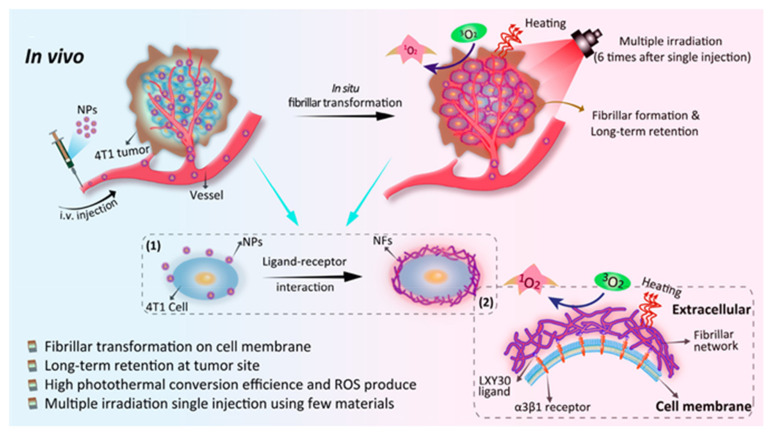
Schematic diagram of transformable peptide nanomaterials with enhanced phototherapeutic effects against cancer. Adapted with permission from [61]. Copyright 2021 American Chemical Society.

In general, the self-assembled nanostructures are spherical. Spherical nanoparticles are easy to prepare, while other shapes of nanomaterials require more structural support and theoretical requirements. In fact, the shape of nanoparticles also plays a key role in drug delivery and release [55,56]. Nanorods were found to be more easily absorbed by cells than spherical nanoparticles, but these materials generally lack targetability [57]. In 2017, Han et al. used a specific peptide sequence—dimethyl maleic anhydride (DMA) modified (Ala-Glu-Ala-Glu-Ala-Lys-Ala-Lys)_2_(AEAEAKAKAEAEAKAK)—to form different shapes of nanoparticles under different pH levels [58]. This drug delivery system with tumor microenvironment-responsive shape switches achieved a therapeutic effect and real-time monitored drug release with the assistance of a photosensitizer. Similarly, in 2019, Zhang et al. reported a dual-targeted nanorod that achieved nuclear targeting by escaping from lysosomes [59]. Recently, He et al. developed an innovative peptide-based nanoparticle to realize light-triggered nitric oxide (NO) release and structural transformation for simultaneously enhanced intratumoral retention and sensitizing PDT [60]. In this work, a peptide with the sequence of Lys-Leu-Val-Phe-Phe (KLVFF) tended to form a β-sheet structure, which was key to structural transformation. Besides delivering therapeutic agents into the cell, it can also achieve in situ reassembly after targeting the tumor cell membrane. A smart peptidic nanophototherapeutic agent was recently reported, which transformed into nanofibers on the surface of the cell membrane for prolonging the retention time in local tumors and improving the effect of phototherapy (Figure 3) [61]. In conclusion, no matter which particular polypeptide sequence was used for self-assembly, it played an important role in structural transformation.

Cancer cells overexpressing enzymes can facilitate the controllable release of drugs, amplifying the therapeutic effect of drugs [62]. Kim et al. have developed visible-light-induced apoptosis activatable nanoparticles of the photosensitizer (Ce6)-caspase-3 cleavable peptide (Asp-Glu-Val-Asp, DEVD)-anticancer drug monomethyl auristatin E (MMAE) conjugate [63]. The activated caspase-3 successfully cleaved DEVD to release the toxic MMAE and produce reactive oxygen species (ROS) under light irradiation to induce severe cytotoxicity. Later, Kim et al. used a similar strategy to design visible-light-triggered prodrug nanoparticles (LT-NPs), in which the self-assembling building blocks were composed of a photosensitizer (verteporfin, VPF), cathepsin B-specific cleavable peptide (FRRG) and doxorubicin (DOX) [64]. LT-NPs specifically were cleaved into VPF and DOX in cancer cells to induce cancer-specific cytotoxicity and ICD under visible light irradiation. Surprisingly, the combination of chemotherapy and PDT enhanced checkpoint blocking of cancer immunity. In terms of immunotherapy, Zhao et al. reported a self-assembling selenopeptide containing a matrix metalloproteinase-2 (MMP-2) enzyme-cleavable linker (PLGVR) [65]. The selenopeptide has properties such as enzyme-induced size reduction and ROS-driven selenization, which delivered therapeutic agents such as DOX, activating NK cells in a programmed manner. At the same time, DOX-induced chemotherapy and selenopeptide-induced immunotherapy can synergistically improve the anti-tumor efficacy.

**Table 1:** Peptide-based assembly for theranostic applications.

Name	Target	Theranostic Type	IC_50_ (Cell Line)	In Vitro or In Vivo	Ref.
G_7_CCERGDS	Cancer cell	PDT	-	In vivo	[45]
antiCD3-G7-RGD	T cell and Cancer cell	Immunotherapy	-	In vitro	[46]
DBT-2FFGYSA	Cancer cell	Imaging and immunotherapy	38.3 μM (PC-3)	In vivo	[47]
NBD-FFYK-TPP	Mitochondria	Imaging and mitochondrial dysfunction	200 μM (HeLa, HepG2, T98G, MCF7)	In vitro	[49]
Mito-FF(ff)-pyrene	Mitochondria	Mitochondrial dysfunction	4–10 μM (HeLa)	In vitro	[50,51]
PEAK-DMA	Cancer cell	PDT	-	In vivo	[58]
TPP-RRRKLVFFK-Ce6	Mitochondria	PDT	-	In vivo	[60]
LXY30-KLVFFK(Pa)	Cell membrane	PTT and PDT	-	In vivo	[61]
Ce6-DEVD-MMAE	Tumor tissues	Light-induced apoptosis	9–10 nM	In vivo	[63]
VPF-FRRG-DOX	Cancer cell	Chemotherapy and PDT	0.54 μM	In vivo	[64]
Sec(Dod)_2_KGPLGVRGRGD	Tumor	Chemoimmunotherapy	0.51 μM (MDA-MB-231)	In vivo	[65]

## 4. Amphiphile Molecule-Based Assembly for Theranostic Applications

Amphiphilic molecules, such as lipid bilayer membranes and proteins, are widely found in living organisms and play an important role in maintaining the normal functions of the body. [66]. Both designing drugs to be amphiphilic and packaging drugs with amphiphilic materials have become common ways to modify drugs to amplify their functions. [67]. Amphiphilic molecules have an inherent characteristic to form a supramolecular nanostructure through self-assembly with size and shape in an aqueous solution [68]. With similar properties, therefore, bioactive amphiphiles will play an irreplaceable role in the preparation of smart drugs with self-assembly behavior (Table 2).

**Figure 4:**
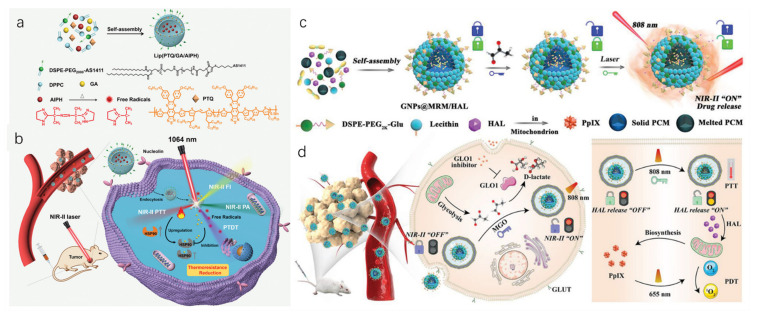
(**a**) Synthesis of NIR-II light excitation Lip (PTQ/GA/AIPH) nanoparticles for fluorescence imaging/photoacoustic imaging-guided combination therapy. (**b**) Schematic diagram of NIR-II light excitation FI/PAI imaging-guided photothermal therapy (PTT) and photonic thermodynamic therapy (PTDT) for triple-negative breast cancer in vivo. (**c**) Synthesis of the GNPs@MRM/HAL nanoparticles and illustration of their “dual lock-and-key” properties. (**d**) Schematic diagram of GNPs@MRM/HAL nanoparticles in NIR-II fluorescence imaging-guided phototherapy of cancer. Adapted with permission from [73]. Copyright 2021 John Wiley and Sons. Adapted with permission from [74]. Copyright 2022 John Wiley and Sons.

Liposomes, which have a similar structure to cell membranes, are widely studied as a means of transporting drugs [69,70]. Several drugs, such as paclitaxel injection and DOX hydrochloride liposome injection, have been approved for clinical use with the assistance of this technology [71]. Liposome drug delivery systems can reduce drug toxicity, increase drug target aggregation, and improve drug efficacy [72]; moreover, it is an efficient strategy to introduce near-infrared (NIR) fluorescent dyes to improve transportation efficiency. In 2021, a NIR-II light excitation phototheranostic nano-medicine was constructed by encapsulating semiconducting polymer, HSP inhibitor, and azo compound into liposomes (Figure 4a,b) [73]. Under the NIR-II laser irradiation, this strategy achieved NIR-II fluorescence/photoacoustic dual-modal imaging-guided photothermal therapy (PTT) and photonic thermodynamic therapy (PTDT), enabling precise diagnosis and effective suppression of tumors with negligible side effects. In a recent study, Xu and coworkers designed a methylglyoxal (MGO)-activatable NIR-II fluorescence probe, which was co-encapsulated with an endogenous photodynamic prodrug into lipid nanoplatforms for NIR-II fluorescence imaging-guided cancer phototheranostics (Figure 4c,d) [74]. Under different light conditions, it activated fluorescence imaging and targeted mitochondria to produce ROS. The two studies above used similar strategies to achieve local tumor imaging and multifunctional tumor suppression. In addition to using liposomes to encapsulate a photosensitizer, researchers are also trying to modify the carrier with additional functions, such as coupling it with anticancer drugs to enhance its therapeutic effect. Lai et al. developed a supramolecular self-assembly strategy to construct a nanodrug based on the host–guest interaction between β-cyclodextrin and diamondol, enabling the co-delivery of *cis*-platin and IR780 [75]. After entering the cell, the nanodrug disintegrates and releases IR780 and *cis*-platin which exert therapeutic effects in the mitochondria and nucleus, respectively. Under the irradiation of an 808 nm laser, the fluorescence of IR780 can cover mitochondria well, and it can generate heat and ROS, resulting in mitochondrial damage and significant inhibition of intracellular ATP synthesis. On the other hand, it also enhances the chemotherapy effect of *cis*-platin.

For anticancer drugs, targetability is always an important topic, since improved targeting can better enrich drugs in tumor tissues and reduce systemic toxicity [76]. Lactose is appropriate as a part of amphiphilic drugs due to its low molecular weight and targeting function [77]. In 2016, Yan and coworkers reported a new kind of drug amphiphile which used lactose and DOX as the targeting ligand and chemotherapy drug, respectively, with the amphiphiles exhibiting enhanced anticancer activity and weak side effects [78]. Recently, employing galactose as a receptor to target tumor cell membranes, Wang et al. designed a kind of acid-responsive polygalactose-co-polycinnamaldehyde polyprodrug (PGCA) which could self-assemble into stable nanoparticles for the delivery of photosensitizer pheophorbide A [79]. In this way, the inhibition of tumor cells can finally be achieved through multi-drug combined PDT, providing new ideas for targeted tumor therapy and multi-way killing of tumor cells. In addition to the delivery of DOX and photosensitizers, the use of hydrophilic lactose to deliver platinum drugs to tumor cells is also a wise choice. Zhou and coworkers reported the design of photoactivatable platinum (IV) (Pt(IV)) amphiphiles containing one or two hydrophilic lactoses for an all-in-one nanomedicine [80]. In this case, platinum drugs released by photoirradiation targeted the nucleus to achieve anticancer effects, while the Cy7.5 on Pt (IV) core enabled temporal and spatial monitoring of the process.

Probes or photosensitizers with hydrophilic or hydrophobic properties can be harnessed to prepare self-assembled prodrugs. In this way, the uptake efficiency of drugs with low absorption will be improved and it may generate a synergistic effect between therapeutic agents. Meanwhile, the real-time monitoring of drug action can be well achieved by introducing a photosensitizer or fluorescent probe. This strategy can better enable the integration of diagnosis and treatment. Yan and coworkers have referenced this concept and designed an amphiphilic drug–drug conjugate (ADDC) based on the hydrophilic anticancer drug irinotecan (Ir) and the hydrophobic anticancer drug chlorambucil (Cb) via a hydrolyzable ester linkage [81]. Remarkably, due to the strong blue fluorescence property of the self-assembled Ir−Cb ADDC nanoparticles (NPs) in aqueous solutions, the real-time tracking of the action site of Ir−Cb ADDC NPs was achieved. To make drugs have a better therapeutic effect, considerable effort has been made to combine bioactive molecules with different functions to produce a synergistic anti-tumor effect, but the resulting drug resistance has become a problem for this strategy. In addition, a class of amphiphilic Gemini iridium (III) complexes (GIC) was synthesized and characterized [82]. Cell-based assays indicate that the drug exhibits outstanding photostability and phototoxicity with satisfactory performance in mitochondria-targeted imaging and PDT effect on tumor cells. The expression of large genes can be regulated by a small piece of the nucleic acid molecule, which is called gene silencing. However, nucleic acids are not easy to enter into cells. Finding a suitable way to deliver them to cells would ensure their therapeutic effect [83]. Zhang et al. proposed the conjugation of small interfering RNA with hydrophobic anticancer drugs to construct precise small-molecule drug-oligonucleotide conjugates with amphiphilic characteristics, and further prepared a nanodrug delivery system based on self-assembly without the carrier [84].

As an alternative to amphiphilic-based self-assembly mentioned above, a class of materials widely used in solar cells, the donor and acceptor unit-based small molecule, has recently been applied to cancer phototheranostics by adding embedded materials to form assembled nanoparticles [85,86]. In 2020, the Ling group designed a donor–acceptor–donor (D–A–D) conjugated small molecule (IID-ThTPA), with isoindigo (IID) as the selective acceptor and triphenylamine (TPA) as the donor, to achieve a high PTT/PDT performance with a superior tumor cooperative eradicating capability [87]. Using Pluronic F127 as an encapsulating matrix to increase the hydrophilic group, these designed molecules were aggregated and formed nanoparticles with good biocompatibility and colloidal stability. Furthermore, IID-ThTPA nanoparticles (IID-ThTPA NPs) exhibit not only competitive photothermal conversion efficiency (35.4%), but also a dramatically high singlet oxygen quantum yield (84.0%). The IID-ThTPA NPs have a good therapeutic effect and show great potential for clinical application. Almost at the same time, the same group reported another D–A–D type small molecule nanoparticle (CSM0-2) with a different number of thiophenes to bridge electron-rich triphenylamine (TPA) and electron-deficient benzo[1,2-c:4,5-c’]bis([1,2,5]thiadiazole) (BBT) [88] Among them, CSM2 showed a broad absorption covering 700–1200 nm with photoacoustic imaging, with a high photothermal conversion efficiency of 31.6% under 1064 nm laser irradiation. Furthermore, CSM2 had good photoacoustic imaging-guided PTT performance in HuH-7 tumor-bearing mice. On the basis of the realization of dual phototherapy, including PDT and PTT, improving the targeting ability to more efficiently target cancer cells can improve the efficiency of drugs and make them effectively accumulate in lesion sites. He et al. designed a kind of CPDT in which they modified the embedding agent to achieve folate functionalization for better targeting [89]. The prepared nanoparticles endowed it with the active targeting of cancer cells, showed good photostability, and enhanced tumor accumulation. In vivo experiments showed that it had a good anticancer effect with no obvious damage to normal tissues. Iridium (III) complexes are emerging as effective bioimaging probes and photosensitizers for phototheranostics due to their excellent photophysical properties. [90,91,92]. However, the absorption of most iridium (III) complexes is located in the visible light region, leading to its limited light penetration depth that is very unfavorable for clinical translations. Zhao et al. constructed a donor–acceptor–donor structure-based iridium (III) complex (IrDAD), which self-assembled as a nanoparticle system (IRDAD-NPs) with good solubility with the help of polyethylene glycol (PEG) [93]. In cancer phototherapy, IRDAD-NPs preferentially accumulate in tumor areas and show significant tumor suppressor effects, with a 96% reduction in tumor volume or even elimination.

**Table 2:** Amphiphile molecule-based assembly for theranostic applications.

Name	Target	Theranostic Type	IC_50_ (Cell Line)	In Vitro or In Vivo	Ref.
Lips(PTQ/GA/AIPH)	Cancer cell	Imaging and PTT, PTDT	-	In vivo	[73]
GNPs@MRM/HAL	Tumor	Imaging and PDT	-	In vivo	[74]
IR780@Pt NPs	Mitochondria	Chemophototherapy	1.2–2.3 μM (143B)	In vivo	[75]
Lac-DOX NPs	Tumor	Chemotherapy	-	In vivo	[78]
PGCA@PA NPs	Cancer cell	PDT and Immunotherapy	-	In vivo	[79]
M(Pt)/V(Pt)	Cancer cell	Imaging and photoactivatable therapy	20.1 μM (HepG2) 55.9 μM (HeLa) 62.5 μM (A549)	In vivo	[80]
Ir-Cb ADDC NPs	Tumor	Chemotherapy	13–15 μM	In vivo	[81]
[Ir(ppy-R)_2_Cl]_2_	Tumor	Imaging and PDT	1.2–1.3 μM (HepG2 and MCF-7)	In vivo	[82]
PTX-DTM-DBCO-chemogene	Tumor	chemo/gene therapy	>10 μM (HeLa)	In vivo	[84]
IID-ThTPA NPs	-	PTT and PDT	-	In vivo	[87]
TPA-BBT	-	PTT	-	In vivo	[88]
FA-PEG-PBLA	Cancer cell	PTT and PDT	-	In vivo	[89]
IrDAD-NPs	Tumor	PDT and PTT	7.1–14.4 μM (A549)	In vivo	[93]

## 5. AIEgen-Based Assembly for Theranostic Applications

Encapsulating traditional fluorescent dyes into the core of nanoparticles often suffers from a problem of fluorescence quenching, which is well known as aggregation-caused quenching (ACQ) [94]. In 2001, Tang developed a novel category of fluorophores with AIE characteristics [95]. This class of fluorescent molecules has no or weak fluorescence emission in solution, but they produce strong fluorescence emission in the aggregated or solid state. This phenomenon is now coined AIE. The beneficial effects of AIEgens for biology have been widely explored; they display unique properties of less false positive signals and larger target-to-background ratios, allowing AIEgens to simultaneously accomplish cancer diagnosis and therapy (Table 3) [96,97].

**Figure 5:**
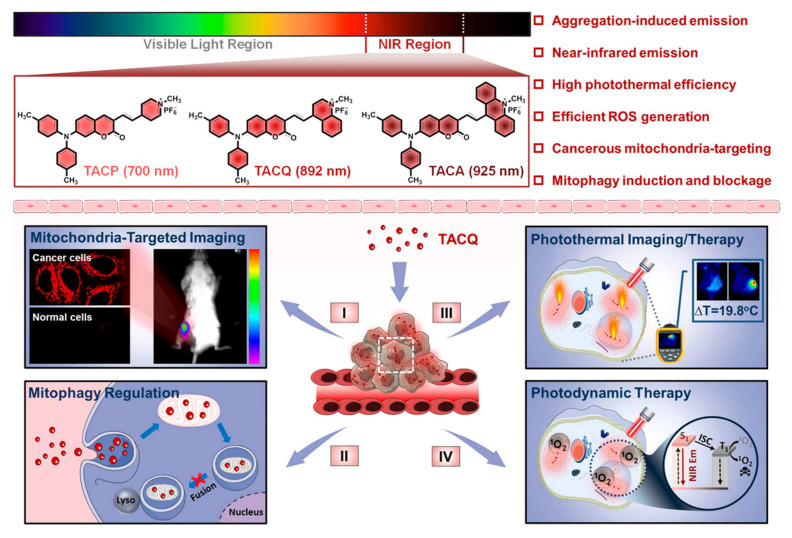
Schematic diagram of NIR AIEgens’ structures and their applications for fluorescence imaging-guided synergistic CT, PTT, and PDT. Adapted with permission from [102]. Copyright 2021 American Chemical Society.

In 2014, Liu and coworkers developed a mitochondria-targeting AIEgen that can induce cell stress and lead to mitochondrial dysfunction for killing cancer cells, and in which the fluorescence of the probe can only be turned on when the rotation around the N-N bond is restricted and the intramolecular hydrogen bonds are formed [98]. Before this work, AIEgens were extensively used for fluorescence imaging, so it offers new possibilities for the development of potential therapeutic agents for diagnosis and therapy. In 2016, Zhang et al. synthesized a series of nanoparticles assembled from pyridinium-substituted tetraphenylethylene salts (PTPE) for targeting mitochondria, and some of them exhibited efficient tumor accumulation and growth inhibition in vivo with negligible systemic toxicity [99]. The assembly of PTPE salts with different alkyl chain lengths and counter anions under different conditions was further explored [100], and the targetability and imaging ability of mitochondria are ascribed to AIE effects, which are promising for cancer therapy. In a recent study, Su et al. developed AIEgens for the treatment of *cis*-platin-resistant cancer cells for the first time, which can induce ROS production and disrupt the mitochondrial structure, impairing mitochondrial and glycolytic metabolism [101]. Chemotherapeutic agents with AIE properties and anticancer activity offer a promising platform for integrating diagnosis and treatment. Recently, Tang et al. synthesized and developed an AIE-active NIR fluorophore with mitophagy-modulating activity for multimodal cancer theranostics (Figure 5) [102].

**Figure 6:**
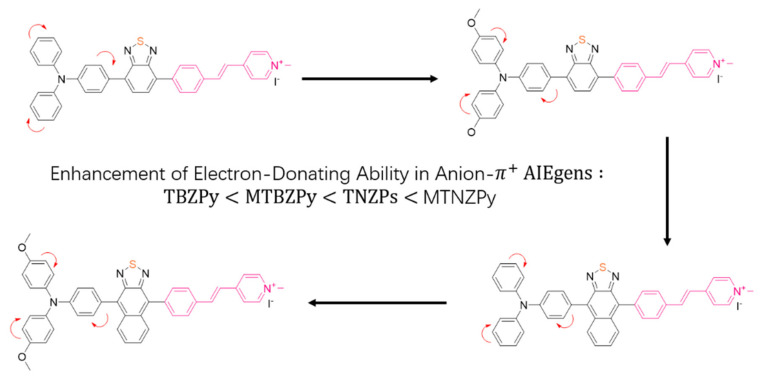
Chemical structures of the final products (TBZPy, MTBZPy, TNZPy, and MTNZPy). Adapted with permission from [106]. Copyright 2020 John Wiley and Sons.

Combing both fluorescence imaging and PDT, phototheranostics has been recognized as a powerful means of cancer treatment [103] In recent years, researchers have begun to extensively explore the use of AIEgens in phototherapy and made significant progress [104]. In 2019, a kind of time-dependent fluorescence-guided PDT was first reported, which employed an AIE-based organic salt photosensitizer for achieving ordered and multiple targeting by simply varying the external conditions [105]. Overcoming the high cost of synthesis and the complex modifications for targeting, the authors used a simple method to obtain AIEgens, which was manipulated to simultaneously achieve AIE fluorescence and aggregation-induced ROS production. When the incubation duration was prolonged, cancer cells were ablated efficiently, leaving normal cells unaffected essentially. In 2020, Tang et al. developed a feasible molecular engineering method to increase the transformation of free radical ROS generators from ^1^O_2_ species for a better PDT effect, which was achieved by efficient electron capture of excited photosensitizers (Figure 6) [106]. In the same year, Tang et al. designed and synthesized D−A conjugated small molecules, which were used as nanoreagents for enhanced PDT guided by photoacoustic imaging [107]. Recently, Tang et al. also came up with new designs and tried to develop an AIEgen that can activate photodynamic and photothermal synergistic therapy (PDT-PTT) upon laser irradiation [108]. This platform enabled one-for-all phototheranostics, which only required one injection and irradiation to achieve excellent tumor removal.

**Figure 7:**
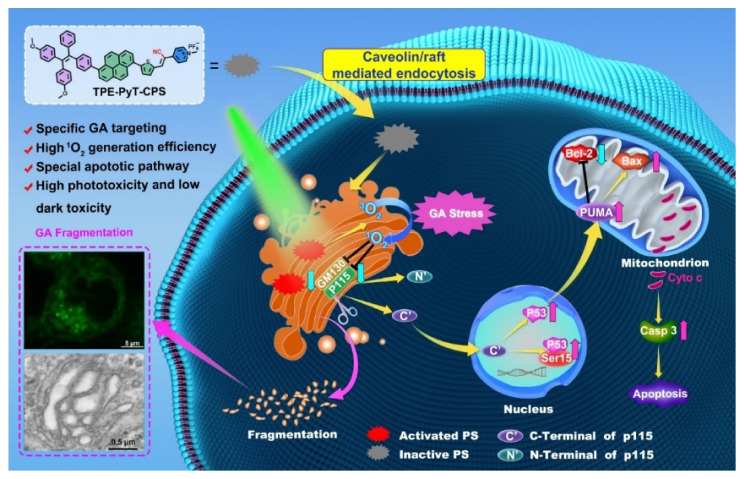
Schematic illustration of AIEgen inducing Golgi stress and the crosstalk between Golgi and mitochondria for cell apoptosis upon PDT. Adapted with permission from [112]. Copyright 2022 Springer Nature.

Besides the efficacy, increasing attention is also paid to improving the targeting, as well as precisely controlling AIE phototheranostic function. The strategy of exploiting the curative effect after photoactivation can exert the effect of phototherapy more accurately and efficiently. In addition to mitochondria, researchers are already trying to target other organelles and combine them with AIEgens to make the most of their molecular features [109]. Ding et al. covalently linked the new AIEgen to a peptide FFKDEL, an endoplasmic reticulum-targeting sequence, to obtain the endoplasmic reticulum-targeting AIEgen [110]. In 2020, Tang et al. designed three molecules with AIE properties (TFPy, TFVP, and TPE-TFPy) to specifically anchor to mitochondria, cell membranes, and lysosomes, respectively, simultaneously obtaining multiple ROS sources from multiple organelles [111]. TPE-TFPy formed nano-sized aggregates in cell culture media, and in situ generated aggregates which internalized into a lysosome of HeLa cells. Without additional biological molecules, a precise PDT effect was achieved only by slight structural adjustment or the introduction of targetable groups. Guo et al. designed AIEgen-based photosensitizers (TPE-PyT-CPS) for targeting Golgi, and they effectively suppressed tumor cells through PDT-induced Golgi oxidative stress [112]. The experimental results showed that the rod-like aggregates were formed in aqueous solution, which was also observed in cell culture medium. Structure–property relationship studies have indicated that the pyrene group contributes to the fast intersystem crossing (ISC) rate and high ^1^O_2_ generation capacity (Figure 7). Liu et al. have reported a kind of membrane anchoring photosensitizer (TBD-RPS) with AIE characteristics [113]. It displayed a typical AIE characteristic of weak emission in the molecular state and strong red emission in the aggregate state. Upon light irradiation, cytotoxic ROS were produced in situ, resulting in direct membrane damage and superior cancer cell ablation.

**Table 3:** AIEgen-based assembly for theranostic applications.

Name	Target	Theranostic Type	IC_50_ (Cell Line)	In Vitro or In Vivo	Ref.
AIE-mito-TPP	Mitochondria	Imaging and PDT	-	In vitro	[98]
DP-PPh_3_, TPE-PPh_3_	Mitochondria	PDT	1.25 μM 3.60 μM (A549R)	In vivo	[101]
TACQ	Mitochondria	Imaging and PDT/PTT	-	In vivo	[102]
4TPA-BQ	Bacteria and cancer cells	PDT	-	In vivo	[105]
TNZPy, MTNZPy	Mitochondria and lysosomes	PDT	-	In vivo	[106]
TTT-1,2,3,4	-	Imaging and PDT/PTT	-	In vivo	[108]
TFPy, TFVP, TPE-TFPy	Mitochondria, cell membrane, and lysosomes	PDT	1.40 μM (4T1) 2.72 μM (HeLa)	In vitro	[111]
TPE-PyT-CPS	Golgi apparatus	PDT	170 nM (HeLa)	In vitro	[112]

## 6. Conclusions and Perspectives

Small molecule-based self-assembly is emerging as a versatile strategy for cancer imaging and targeted therapy, showing the advantages of their unique all-in-one capacity, longer retention time, overcoming of drug resistance, and better therapeutic performance, etc. In this review, we mainly focused on summarizing and discussing the advances in small molecule-based self-assembly for cancer theranostics based on three types: peptides, amphiphilic molecules, and AIEgens. Peptide sequences are successfully functionalized by targeted molecules, organic dyes, and/or bioactive molecules for targeted therapy of cancer in vitro and/or in vivo, while the introduction of an environmentally sensitive dye to peptides enables the monitoring of their assembly behaviors, which is also appliable for diagnosis. Considerable advances have also been obtained in amphiphilic small molecule-based self-assembly for theranostics, in which functional small molecules assemble or co-assemble with other functional molecules for multiple purposes, such as fluorescence imaging (NIR-I, NIR-II), photoacoustic imaging, PDT, PTT, and/or PTDT. AIEgens were also well harnessed for fluorescence imaging and phototherapy based on their desirable photophysical properties, with the assistance of self-assembly. These advances have resulted in accelerating the implementation of self-assembled biomaterials for in vivo studies and even further clinical use.

The advanced examples above give us faith for further exploring small molecule-based self-assembly for biomedical applications. However, it should also be noted that there are still some challenges for their practical use. Presently, it is still hard to achieve precisely controllable assembly or reassembly of small molecules, and the precise relationship between small molecules’ structures and their assembly behaviors is not clear. We note that advances in computational modeling and simulation have allowed in silico studies of the self-assembly of small molecules. Further intensive use of these techniques will deepen the understanding of the assembly and regulation mechanism of small molecules’ self-assembly, providing huge opportunities for the rational design of self-assembled small molecules for cancer theranostics. At the same time, as self-assembly depends on non-covalent interaction, complete assembly of small molecules often requires a high concentration, which may cause potential cytotoxicity. Moreover, it is sometimes difficult for cellular assembly of small molecules due to the requirement of high concentrations of small molecules in a local site in celluo or in vivo. Therefore, we need to find more suitable materials with low critical micelle concentrations. To our delight, the self-assembly field has received increasing attention from scientists in materials, chemistry, physics, biology, and medicine. We believe clinics will benefit from small molecule-based self-assembled biomaterials once a deeper understanding of them has been reached in the near future.

## Data Availability

Not applicable.
